# More meat on the bone: Perioperative nutrition and elective orthopaedic surgery

**DOI:** 10.1113/EP092872

**Published:** 2025-05-31

**Authors:** Brendan Egan, Brian M. Devitt

**Affiliations:** ^1^ School of Health and Human Performance Dublin City University Dublin Ireland; ^2^ Florida Institute for Human and Machine Cognition Pensacola Florida USA; ^3^ UPMC Sports Surgery Clinic Santry Demesne Dublin Ireland

**Keywords:** atrophy, protein, rehabilitation, skeletal muscle, supplementation

1

The World Health Organization defines healthy ageing as ‘the process of developing and maintaining the functional ability that enables wellbeing in older age’, with the importance of skeletal muscle contributing to that functional ability now widely appreciated. This definition also implies a life course perspective to healthy ageing, such that factors that influence both development (early life) and maintenance (middle to older life) of functional ability and, by extension, articular joint function, skeletal muscle mass and strength, warrant attention. Although there is some inevitability to declines in joint health, skeletal muscle mass, and strength with advancing age, the greatest threat to healthy ageing from the muscle‐centric perspective is episodes of accelerated loss and incomplete recovery, often referred to as the ‘catabolic crisis model’ (English & Paddon‐Jones, 2010). In that model, these episodes punctuate the normal ageing process as a consequence of acute illness or injury, leading to mandatory hospitalization that coincides with bed rest or forced inactivity. The resultant unloading and disuse of skeletal muscle, even when short term, can have profound effects on functional ability, metabolic health and immune function, such that mitigation strategies during unloading, and/or augmentation approaches during recovery and rehabilitation, are of great importance and have broad healthcare implications. In this issue of *Experimental Physiology*, Witard et al. (2025) provide a narrative review focused on the topical issue of perioperative nutrition interventions as mitigation strategies around orthopaedic surgery, and as augmentation strategies during rehabilitation, for outcomes related to skeletal muscle mass and function.

Elective orthopaedic surgery, such as total hip arthroplasty (THA), total knee arthroplasty (TKA) and anterior cruciate ligament reconstruction (ACLR), presents the typical inflammatory, catabolic and hypermetabolic states associated with surgery. In older adults, orthopaedic surgery usually also requires short‐term bed rest and reduced physical activity in the days after surgery. As a result of the combined effects of catabolism and immobilization, these surgeries consistently result in skeletal muscle atrophy and declines in physical function in the postsurgical period. Moreover, arthrogenic muscle inhibition after surgery can reduce muscle activation during contraction and lead to a lesser stimulus received by the contractile tissue during rehabilitation exercises that target restoration of skeletal muscle mass and function.

Given the recognized role of muscle protein turnover (synthesis and breakdown) in the regulation of muscle mass, Witard et al. (2025) focus largely on essential amino acid and protein supplementation after surgery, for which the evidence to date, albeit somewhat limited, does suggest some effectiveness on these outcomes in patients who have undergone THA, TKA, surgical treatment of hip fracture or ACLR. The scarcity of studies in this domain has been highlighted recently by a systematic literature review identifying only 14 studies (611 patients; 224 males and 387 females) that have explored this question in THA (*n* = 3), TKA (*n* = 5), surgical treatment of hip fracture (*n* = 3) and ACLR (*n* = 3) (George et al., 2023).

In a broader context, perioperative care includes preoperative, intraoperative and postoperative phases. The postoperative phase describes the period of hospital stay up to discharge (usually days) but can extend longer (weeks and months) into recovery depending on the surgery. Appropriately prescribed exercise‐based rehabilitation remains *sine qua non* for complete recovery from elective orthopaedic surgery. Studies focusing on postoperative nutrition and outcomes assessed after weeks or months of intervention encompass the interaction between nutrition and exercise‐based rehabilitation (George et al., 2023; Witard et al., 2025), such that outcomes can also reflect the effect of nutrition interventions to augment adaptation to rehabilitative exercise training.

In contrast to those longer‐term muscle‐centric outcomes, from a surgical perspective, the immediate postoperative concerns relate to wound healing, delayed early mobilization, risk of falls, periprosthetic fracture and prolonged hospital stays. Poor nutrient status and malnutrition are common perioperative comorbidities that affect many older adult patients and are strongly associated with worsened postoperative outcomes and higher healthcare costs (Aziz et al., 2025). Interventions that improve dietary intake and nutrient status during hospital stays could improve short‐term postoperative outcomes, in addition to quality of life later in recovery, and thereby reduce overall healthcare costs for patients and health systems. Unsurprisingly, the most recent National Institute for Health and Care Excellence (NICE) guidelines on perioperative care specifically identify the need for research on clinical outcomes and cost‐effectiveness of enhanced recovery programmes in the postoperative period (NICE, 2020).

Yet, given the impact of poor nutrient status and malnutrition, the potential for nutrition intervention in the preoperative phase also warrants strong consideration. In addition to the immediate (<12 h) preoperative period, this phase includes a pre‐assessment for the patient that is usually weeks before elective orthopaedic surgery. Screening for nutrient status and malnutrition risk at this time point can allow for early intervention. Therefore, in its broadest remit, perioperative nutrition interventions should encompass much more than postoperative protein supplementation or oral nutrition solutions, and instead can also include focus on optimizing dietary intake prior to surgery. This approach would require addressing nutrient deficiencies known to have greater likelihood in older adults (e.g., protein, calcium, vitamin D and vitamin B) because of the age‐related changes (e.g., physiological, sensory, cognitive, social) that increase risk factors contributing to the development of deficiencies in these patients.

Even as the evidence for pre‐, peri‐ and postoperative nutrition intervention accumulates, there remains the perennial challenge of the evidence‐to‐practice gap in implementing evidence‐based, interdisciplinary, multicomponent nutrition care. For patients, although many explicitly perceive nutrition to be of importance to postoperative outcomes and state that they would modify their habits to improve outcomes, most patients do not know how to optimize dietary intake or do not receive adequate instruction on how to do so. Perioperative nutrition interventions therefore require the support of dietitians or appropriately qualified registered nutritionists, which, in itself, adds additional economic burden on healthcare systems. Yet as Witard et al. (2025) starkly highlight, well over 100 000 surgeries for THA and TKA are performed each year in the UK, with these surgeries costing tens of thousands of pounds each. In that context, economic evaluations of cost‐effectiveness of nutrition interventions across all phases of the perioperative period might result in a strong case for greater investment in perioperative nutritional support. However, before such evaluations are performed and greater investment provided, further research is needed to establish a broader evidence base for perioperative nutrition, specifically in middle‐aged and older adults undergoing elective orthopaedic surgery. That research could have important implications for improving recovery after orthopaedic surgeries and thereby provide economic value to healthcare systems and direct benefits to patients by improving postoperative outcomes and reducing long‐term burden.

## AUTHOR CONTRIBUTIONS

Both authors have read and approved the final version of this manuscript and agree to be accountable for all aspects of the work in ensuring that questions related to the accuracy or integrity of any part of the work are appropriately investigated and resolved. All persons designated as authors qualify for authorship, and all those who qualify for authorship are listed.

## CONFLICT OF INTEREST

None declared.

## FUNDING INFORMATION

None.
